# Determinants of mental and financial health during COVID-19: Evidence from data of a developing country

**DOI:** 10.3389/fpubh.2022.888741

**Published:** 2022-08-31

**Authors:** Falak Khan, Muhammad A. Siddiqui, Salma Imtiaz, Shoaib A. Shaikh, Chin-Ling Chen, Chih-Ming Wu

**Affiliations:** ^1^FAST School of Management, Islamabad, Pakistan; ^2^National University of Computer and Emerging Sciences, Islamabad, Pakistan; ^3^Department of Software Engineering, International Islamic University, Islamabad, Pakistan; ^4^Electrical Engineering Department, Sukkur IBA University, Sukkur, Pakistan; ^5^School of Information Engineering, Changchun Sci-Tech University, Changchun, China; ^6^Department of Computer Science and Information Engineering, Chaoyang University of Technology, Taichung, Taiwan; ^7^School of Civil Engineering and Architecture, Xiamen University of Technology, Xiamen, China

**Keywords:** mental health, COVID 19, financial health, financial capability, financial literacy, socioeconomic and demographic factors, developing countries

## Abstract

Mental and emotional issues are the top-level concerns of public health worldwide. These issues surged during Coronavirus (COVID-19) pandemic due to varied medical, social, and personal reasons. The social determinants highlighted in the literature mainly focus on household solutions rather than on increasing the financial wellbeing of individuals, especially for the most vulnerable groups where the psychological distress coming from the social inequalities cannot be entirely treated. Hence, this study attempts to familiarize the financial capability (the financial literacy, attitude, skills and behavior required for effective financial management) construct into public health domain in the times of COVID-19 as a determinant of psychological distress, and also explores the role of gender in it. The study uses Ordinary Least Square (OLS) regression analysis and employs mental distress questions and Organization for Economic Cooperation and Development (OECD) 2018 financial capability toolkit to collect data from a large sample of households from all over Pakistan. It is inferred that the higher the financial capability, the lower the financial and mental distress during COVID-19. Additionally, females are less financially knowledgeable, depict poor financial behaviors, and face more psychological issues than their counterparts. Age and education are also linked to mental stress during COVID-19. Finally, gender plays a moderating role in financial behavior, and financial and mental stress of households. As evident, COVID-19 is not going away soon hence the findings are relevant for policymakers to proactively plan for the pandemic's upcoming waves and help people be better financially equipped to fight against this or any upcoming crisis, and achieve better mental and physical health.

## 1. Introduction

The World Health Organization (WHO) emphasizes on six different components of mental health that include self-complacency, self-rule, self-growth, good relations, purpose in life, and social wellbeing of an individual (1). These emotional and mental issues, being top-level concerns of public health all over the world, have risen significantly during the Coronavirus (COVID-19) outbreak that has brought uncertainties to all walks of life, including the fear of contracting the virus and death from it (2–7). Consequently, people are suffering from anxiety, sadness, nervousness, frustration, and other severe mental disorders (8–10). Studies have also reported suicidal attempts as a result of social distancing, limited physical mobility, lockdowns, quarantines, self-isolation, and financial stress during the current pandemic (11).

Individuals not only had to face health emergencies, but the financial requirements to pay for healthcare, increased utility bills due to work from home, unemployment, and stockpile also surged (12–14). These ambiguous circumstances, the uncertainty of the pandemic situation, and the inability to cope with it, resulted in further financial stress, which is the state of anxiety, worry, and tension related to debt, money management and current expenses (15). Since financial stress is linked to mental health (16), when individuals were not able to fund their obligations, their stress increased, and as a result, their health was affected (17).

It is noted that people with a better understanding of savings and finance are better at financial management, and they are more likely to be prepared for any financial shock, or emergency (18) hence possess better mental health than the ones who do not understand the concept and are more likely to have mental health issues as compared to their counterparts (19, 20). However, whether people have learned from the previous crises, like the “Great Recession”, and have prepared themselves against financial and mental shocks by increasing their financial resilience through positive financial behaviors or not, remains unanswered.

Prolonged financial hardships can have detrimental effects on individuals' wellbeing and health (21–24). Despite the ubiquitous acknowledgement of economic hardship as a determining factor of health, it has mainly been assessed through income disparities, food and housing insecurities and other basic needs (16, 22). Wide-ranging solutions, such as efforts to improve financial knowledge, financial attitude and financial behavior to prepare individuals for financial shocks through savings and decreasing income uncertainty among poor in developing countries, are missing from the field of public health (25, 26). This becomes increasingly important and more relevant in the times of a pandemic like COVID-19, where rise in mental stress levels is repeatedly highlighted in the literature (27–31). Building on the evident link between income and mental health, this study demonstrates that financial capability is a significant, independent social determining factor of mental health which has helped individuals to fight against the worlds' most unprecedented global crisis in the shape of the COVID-19 pandemic, and can be influenced to develop overall health and wellbeing.

This study is organized into 5 sections with literature review and background in Section 2; Section 3 entails the detailed methodology adopted to collect and analyze data. Results are reported in Section 4, whereas Section 5 concludes the study with implications and future recommendations.

## 2. Literature review

Stress in any form has its effects on health, and financial stress is the most common cause of anxiety and depression (1), which is the emotional and mental tension resulting from the inability to meet financial needs like paying for bills, rent, groceries, education for children and healthcare (2). Financial stress can even lead to further varied and complex issues such as delays in health care, as observed in 29% of Americans who neglected their medical issues and postponed medical checkups because of financial constraints, which had a deteriorating effect on their health (32). Financial stress can also result in poor mental and physical health, resulting in headaches, migraines, heart diseases, sleep disorders, diabetes, and much more. Moreover, prolonged financial distress can lead to flaring-up of symptoms of other diseases (33). Anxiety induced by this can also lead to unhealthy coping behaviors, which include increased drug abuse and emotional eating, as 33% Americans were reported to have unhealthy eating behaviors as a consequence of stress (34).

Psychological stress can be a result of many internal as well as external factors that include socioeconomic demographic factors like gender, income, race, region, education, and others (35). It is also postulated that low-income groups may experience high financial stress as their work environments are unsafe and their jobs are less flexible. They cannot even think about switching jobs due to lack of financial support (36). Additionally, these groups have no access to health insurance or health care facility, which adds to their mental and physical health issues (37). Similarly, less educated women are more prone to mental health issues caused by food insecurity and financial distress as compared to their counterparts (38). These issues are faced more by developing countries than developed countries as the unemployment rate is high (39).

Further, lack of comprehensive education is also highlighted as a significant contributor to financial stress. The importance of financial literacy, which refers to money management knowledge and skills to make informed decisions for better financial wellbeing, is even enhanced in the case of a crisis, where due to lack of financial literacy, poor money management, and ill planning, people faced financial distress. (40). It is also noted that individuals with better financial education, financial attitudes, and financial behaviors are better at financial management, and they are more likely to be prepared for any financial shock or emergency and hence, possess better mental health (41).

With the significance of financial knowledge and behavior clearly established for better mental health, its relevance surges during an emergency situation like that of COVID-19, the world's most unprecedented global crisis (42). During COVID-19, many individuals lost their jobs globally, making them unable to fulfill basic needs such as paying rent, buying groceries, and other educational and medical expenditures, consequently affecting the mental and physical health of the masses (17, 30, 42). As a result, in this health and employment crisis case, regaining financial footing became a challenge, and many were found scrambling (43).

Medical research mainly focused on the medical issues and illnesses arising from the COVID-19 pandemic in developed countries (44, 45). However, little is known about the public mental health issues arising from financial distress, and whether people have learned from the previous setbacks such as the “Great Recession”, and have prepared themselves for financial as well as mental shocks in terms of increasing their financial resilience through positive financial behaviors or not during COVID-19 (Table 1 reports the focus and shortcomings of cited work during COVID-19 in a greater detail). Hence, the current study fills this gap by exploring the much ignored role of financial attitude, financial behavior, and financial knowledge on mental stress due to the financial situation of individuals during the current pandemic, and it also studies the role of socioeconomic and demographic factors. Since, the dynamics of financial literacy levels vary along regions and cultures (19), the evidence in this study is presented by taking into account data collected from a developing country, Pakistan.

**Table 1:** Summary of literature on mental health stressors during COVID-19.

**References**	**Sample size**	**Country**	**Focus**	**Shortcomings**
Ali et al. (30)	420	Bangladesh	Financial wealth, financial wellbeing, and economic hardships are tested as determinants of mental stress during COVID-19.	The drivers of financial wellbeing are not considered.
Cao et al. (31)	430	China	Association of mental health with COVID-19 is investigated with focus on demographics and family support.	Financial stressor is not gauged.
Gloster et al. (17)	9,565	78 Countries	Impact of COVID-19 on only the mental health is tested.	The role of financial stressor remained unaddressed.
Halliburton et al. (46)	1,220	United States	Experience of students is explored by focusing on mental health issues during COVID-19.	Any possible role of financial distress is not tested.
Kuang et al. (47)	2044	India	Awareness of symptoms, risk perception, behaviors and psychological stressors during COVID-19 are tested.	Affect of financial wellbeing is not explored.
Liu et al. (28)	470	United States	Effect of COVID-19 on mental health due to changes in leisure activities is seen.	Financial stress is not considered.
Siddiqui et al. (24)	4,020	Bangladesh	Evidence of correlation between financial difficulties and mental health is investigated.	The deep-down causes of financial difficulties are not considered.

## 3. Methods

The study is conducted by adaption of harmonized Financial Capability Survey (48) available globally by the Organization for Economic Cooperation and Development (OECD). The survey was divided into questions based on three parts: financial knowledge, financial attitude, and financial behavior, that are clubbed together to form the overall financial literacy of households (49). This section discusses the methods of construction of variables and data collection.

Financial knowledge helps households make well-informed financial decisions by comparing different products; it also facilitates them to employ numeracy skills (which can be helpful to understand financial news and events, thus facilitating people to respond in an appropriate manner). Its score is computed based on seven questions about the concept of inflation, interest compounding, and risk and return. The aggregate score ranges from 0 to 7.

Financial behavior concerns taking such financial actions that result in financially savvy individuals paying bills on time, shopping around to make informed purchases; hence its aggregate score is based on questions relating to savings, budgeting, borrowing, payments, and purchases and can go from 0 to 9.

Financial attitude measures individuals' attitude toward saving and spending and time preference for example, “I tend to live for today and let tomorrow take care of itself” which also leads to financial resilience. This score ranges from 1 to 5.

The overall financial capability score is the summation of the three sub-scores: financial knowledge, financial attitude, and financial behavior, and can range from 1 to 21. Other than the demographic and socioeconomic factors (gender, marital status, region, education, income, age, and experience), the survey additionally asks whether the respondents are stressed due to their financial situation during COVID-19. The econometric model is as follows:


(1) $$\begin{array}{r} MeCOVID_{i}=\alpha_{0}+\beta_{1}FC_{i}+\beta_{2}X_{i}+\epsilon_{i} \end{array}$$

where *MeCOVID*_*i*_ is the mental stress during COVID-19, *FC*_*i*_is the financial capability score calculated based on OECD toolkit 2018 (48), and *X*_*i*_ is the demographic and socioeconomic variable such as gender, marital status, region, education, income, age, and experience and *epsilon*_*i*_ is the error term.

To check the moderation effect, Process Macro (50) is used by applying the following econometric model.


(2) $$\begin{array}{r} MeCOVID_{i}=\alpha_{0}+\beta_{1}FB_{i}+\beta_{2}Gender_{i}+\epsilon_{i} \end{array}$$

(3) $$\begin{array}{r} MeCOVID_{i}=\alpha_{0}+\beta_{1}FB_{i}+\beta_{2}Gender_{i}+\beta_{3}Gender_{i}*FB_{i}+\epsilon_{i} \end{array}$$

Here *FB*_*i*_ refers to the financial behavior and *Gender*_*i*_ is the dummy variable, where “1” represents female gender.

## 4. Data collection

A sample of 730 (seven hundred thirty) individuals from several households across Pakistan constitutes this study. Convenience sampling technique is used to collect the data as due to the lockdown and government restrictions during the pandemic, physical data collection was very difficult, hence reaching out people was a limitation. The data is collected from all five provinces of Pakistan: Khyber Pakhtunkhwa (KPK), Sindh, Punjab, Gilgit-Baltistan, and Baluchistan. In addition to this, data is also collected from Azad Jammu and Kashmir. The response rate was 80.8%, as 730 questionnaires were received from 904 sent out surveys.

## 5. Results

Table 2 shows the descriptive analysis (means and standard deviation) of the data collected from all over Pakistan. Of all the respondents 51% are male, 48% are married, 38% have received tertiary education, and 48% have received secondary education. The data is distributed among different income groups, with medium-income groups constituting 29% of the data, 38% belonging to middle age groups, and 24% having a work experience of 3–6 years.

**Table 2:** Descriptive statistics.

**Variable**	**Mean %**	**St. dev**
Male	51	0.5
Married	48	0.5
Urban	83	0.38
Intermediate education	15	0.354
Secondary education	48	0.5
Tertiary education	38	0.485
Female	49	0.5
Income below PKR 50,000	58	0.494
Income between PKR 50,000–100,000	29	0.453
Income above PKR 100,000	13	0.341
Age 18–30	52	0.5
Age 30–50	38	0.486
Age 50 and above	10	0.305
Work experience below 3 years	39	0.488
Work experience between 3 and 6 years	24	0.425
Work experience above 6 years	37	0.484

Table 3 reports the the number of people stressed due to COVID-19 with their chi-squared coefficients and their level of significance. It can be observed that females are more stressed than men. In a similar manner, married people are more stressed because of their increased responsibilities. 61% respondents belonging to urban areas are observed to be more worried about their financial situation since these areas are more prone to COVID-19 as compared to rural areas. It is also noted that the education level of people matters, as the ones who were more educated stressed less about their financial situation. High-income respondents are less worried about COVID-19 regarding their financial situation as their income can cover their health costs. Old age groups are more prone to contracting COVID-19; hence they are more worried about COVID-19 and their financial situation respectively. As work experience increases, individuals are more likely to have better income and are less financially stressed.

**Table 3:** Percentage of sample stressed and non-stressed due to COVID-19.

**Variables**	**Stressed due to COVID-19 (%)**	**Not stressed due to COVID-19 (%)**	** ${\tilde{\chi }}^{2}$ **
Male	52.10	47.90	35.9^***^
Female	73.40	26.60	35.9^***^
Married	58.10	41.90	5.69^***^
Urban	60.90	39.10	3.97^***^
Intermediate education	69.20	30.80	2.3^**^
Secondary education	65.50	34.50	2.4^**^
Tertiary education	56.30	43.70	7.43^***^
Income below PKR 50,000	65.30	34.70	3.28^***^
Income between PKR 50,000–100,000	60.50	39.50	0.55
Income above PKR 100,000	55.10	44.90	2.69^**^
Age 18–30	55.60	44.40	6.06^***^
Age 30–50	58.80	41.20	0.71
Age 50 above	68.80	31.20	0.267
Work experience below 3 years	68.90	31.10	7.98^***^
Work experience between 3 and 6 years	63.60	36.40	0.1
Work experience above 6 years	55.10	44.90	10.17^***^

Where

* ,

** , and

*** are 10, 5, and 1% significance levels, respectively.

Table 4 reports the effect of financial capability components on financial distress during COVID-19. It can be inferred that financial knowledge and financial behavior are significantly negatively related to financial distress (significant at 1%), whereas a statistical link with financial attitude could not be established. Additionally, respondents belonging from Punjab, Sindh, and KPK are significantly less worried about their financial situation as compared to those belonging from Balochistan; in a similar manner, females are significantly more worried about their financial situation during COVID-19 (significant at 1%) and hence have faced more mental health issues.

**Table 4:** Financial capability components effect on stress amid COVID-19.

**Variable**	**SE**	**Coefficient**
Financial knowledge score	[0.025]	−0.142^***^
Financial attitude score	[0.075]	−0.029
Financial behavior score	[0.024]	−0.065^***^
Married	[0.110]	0.025
Age 18–30	[0.134]	−0.109
Age 30–50	[0.170]	−0.12
Urban	[0.111]	−0.054
Punjab	[0.228]	−0.531^**^
Sindh	[0.285]	−0.830^*^
KPK	[0.256]	−0.426^***^
AJK	[0.266]	−0.341
Gilgit	[0.361]	−0.098
Experience below 3 years	[1.090]	−0.37
Experience between 3 and 6 years	[1.094]	−0.144
Secondary education	[0.126]	0.008
Tertiary education	[0.137]	−0.035
Income between PKR 50,000–100,000	[0.103]	−0.0482
Income above PKR 100,000	[0.156]	−0.114
Female	[0.091]	0.214^***^
Intercept	[1.158]	5.15^***^
R-Squared	0.134	
Number of observations	730	

Where

* ,

** , and

*** are 10, 5, and 1% significance levels, respectively.

**Figure 1:**
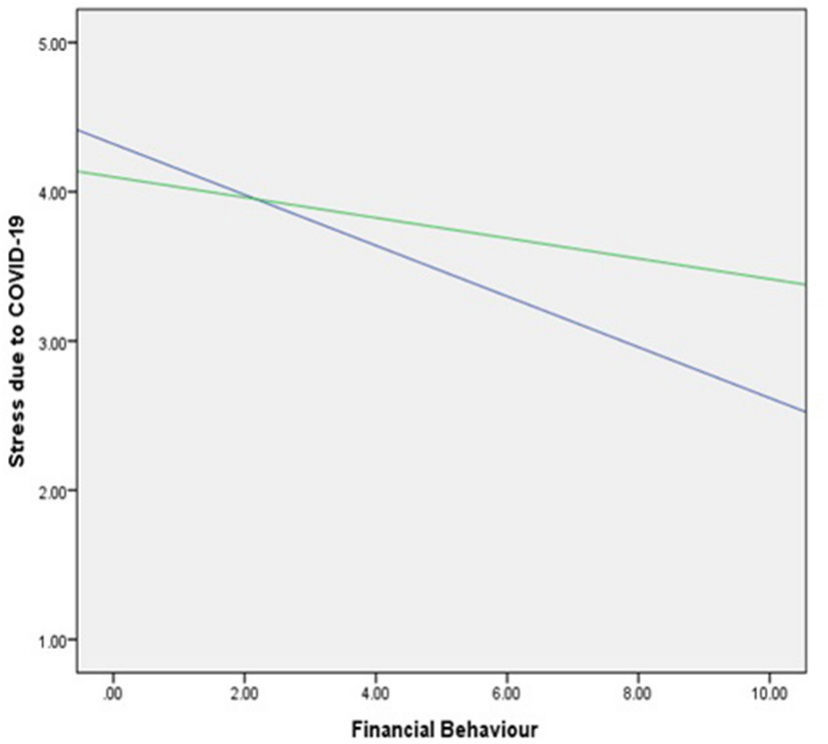
Moderation of gender between COVID-19 distress and financial behavior.

Table 5 reports regression results for the effect of financial capability on financial distress related to COVID-19. The score comprises of three components: financial attitude, financial knowledge, and financial behavior, so individuals with better overall financial capability have well-managed their financial situation and are less stressed. The overall financial capability scores are significantly negatively related to financial distress (significant at 1%). It can be observed from the results reported in Table 6 that gender moderates the relation between financial behavior and stress due to COVID-19. A change in R-square suggests that gender as a moderator affects stress more than an independent variable. On average, the stress levels of females are higher than males, where males have scored 4.1 in the stress levels and females have scored 4.32. This can also be observed from the moderation graph shown in Figure 1.

**Table 5:** Effect of overall financial capability on COVID 19 distress.

**Variable**	**SE**	**Coefficient**
Financial capability score (overall)	[0.013]	−0.096^***^
Married	[0.110]	0.0128
Age 18–30	[0.134]	−0.11
Age 30–50	[0.170]	−0.101
Urban	[0.111]	−0.061
Punjab	[0.228]	−0.513^***^
Sindh	[0.284]	−0.791^***^
KPK	[0.255]	−0.417
AJK	[0.264]	−0.308
Gilgit	[0.360]	−0.04
Experience between 3 and 6 years	[1.094]	−0.236
Experience above 6 years	[1.099]	−0.393
Secondary	[0.125]	−0.009
Tertiary	[0.137]	−0.054
Income between PKR 50,000–100,000	[0.103]	−0.046
Income above PKR 100,000	[0.156]	−0.11
Female	[0.091]	0.222
Intercept	[1.135]	5.519^***^
R-Squared	0.13	
Number of observations	730	

Where

* ,

** , and

*** are 10, 5, and 1% significance levels, respectively.

**Table 6:** Moderation of gender between financial behavior and COVID-19 distress.

		**SE**	**LLCI**	**ULCI**
R-Squared	0.07			
Change in R-Squared	0.0064			
Constant		4.32^***^	3.93	4.703
Financial behavior		−0.17^***^	−0.234	−0.105
Gender		−0.221	−0.73	0.2871
Financial behavior^*^gender		0.101^***^	0.012	0.191

Where

* ,

** , and

*** are 10, 5, and 1% significance levels, respectively.

## 6. Discussion

Psychological stress severely impacts individuals' emotional and mental health, and several factors increase it. For instance, stress and anxiety in any individual are augmented as a result of the inability to fulfill basic needs such as paying the rent, buying groceries, and other educational and medical expenditures, especially during a crisis (51). Those who have a better education tend to be less stressed about their financial situation. High-income respondents are less worried about their financial situation during the pandemic as their income can cover their health costs. Old age groups are more prone to contracting COVID-19. Hence, they are more worried about COVID-19 and their financial situation. Individuals with better or more work experience are more likely to have better income and are therefore less stressed.

The study also incorporated demographics and socioeconomic factors to observe their dynamic role in psychological distress due to COVID-19. It is observed that in line with many studies, women tend to be less financially literate, hence depict poor financial behaviors and are more likely to be stressed due to COVID-19 because of the limited financial access and exposure given to females in developing countries (52–54). Thus, lack of financial literacy also contributes to increased stress levels of women due to the current pandemic. In addition, it is observed that financial attitude does not impact psychological distress during COVID-19 (55).

Similarly, it can be observed that more educated and high-income people show better financial behaviors and are less worried about their financial situations, hence facing a low level of psychological distress (56). The people who possess low education levels or fall under low-income groups are not knowledgeable enough about saving during the crisis, and are more worried about their necessities during an emergency (51). The study also investigated whether people with high debt levels are more worried about their financial distress during the pandemic or not, and it is conceived that households with high debt levels are financially distressed during the pandemic (43).

## 7. Practical implications and future directions

Financial stress is considered as one of the main mental stressors, and in severe cases may lead to increased risk of heart disease and physical pain. This risk may further increase during global crises, disasters, or pandemics like that of COVID-19. Despite evident contribution of efforts made to increase financial capability of individuals in protecting them from financial shocks and building their financial resilience to minimize the income disparities and achieve financial and healthier wellbeing, this remains unaddressed in the public health backdrop. To better understand the implication of financial health on public health, integration of financial capability into other health determinants will help drive far-reaching and effective interventions by offering solutions through a framework which is not based on siloed methods of housing, food, utilities or other economic hardships only. It can help focus on these matters by pursuing to improve financial knowledge and financial behavior of masses especially females, which will lead them to make well-informed financial decisions regarding how to spend, borrow, and save. Additionally, the public health community with the help of policymakers should devise financial literacy programs and create opportunities for the ones who are socially as well as financially marginalized to construct their wealth, bring sustainability and prepare them for any future crisis.

Though the current study is the first of its kind to conduct a financial capability survey in Pakistan, to gauge the effect of stress during the COVID-19 pandemic, it has a few shortcomings. To begin with, though the survey covers all provinces of Pakistan yet, due to limited physical mobility during the current pandemic, data is mainly collected online. Moreover, other reasons for financial distress are not explored in the study. The current study creates a base for future scholars in the area to investigate the impact of financial literacy-based interventions that will build financial capabilities of people to increase their financial wellbeing, that can consequently improve their mental health.

## Data availability statement

The raw data supporting the conclusions of this article will be made available by the authors, without undue reservation.

## Ethics statement

Ethics approval and written informed consent were not required for this study in accordance with national guidelines and local legislation.

## Author contributions

All authors listed have made a substantial, direct, and intellectual contribution to the work and approved it for publication.

## Conflict of interest

The authors declare that the research was conducted in the absence of any commercial or financial relationships that could be construed as a potential conflict of interest.

## Publisher's note

All claims expressed in this article are solely those of the authors and do not necessarily represent those of their affiliated organizations, or those of the publisher, the editors and the reviewers. Any product that may be evaluated in this article, or claim that may be made by its manufacturer, is not guaranteed or endorsed by the publisher.
